# Research barriers in children and young people with life-limiting conditions: a survey

**DOI:** 10.1136/bmjspcare-2018-001521

**Published:** 2018-07-31

**Authors:** Jordana Natalie Peake, Emma Beecham, Linda J M Oostendorp, Briony F Hudson, Patrick Stone, Louise Jones, Monica Lakhanpaul, Myra Bluebond-Langner

**Affiliations:** 1 The Louis Dundas Centre for Children’s Palliative Care, UCL Institute of Child Health, London, UK; 2 Marie Curie Palliative Care Research Department, University College London Division of Psychiatry, London, UK; 3 Pathway, London, UK; 4 Population, Policy and Practice Programme, UCL Institute of Child Health, London, UK

**Keywords:** paediatrics, clinical decisions, ethics, methodological research, symptoms and symptom management

## Abstract

Studies indicate research ethics committee (REC) approval and clinician gatekeeping are two key barriers in recruiting children and young people (CYP) with life-limiting conditions (LLCs) and life-threatening illnesses (LTIs) and their families to research.

**Objectives:**

To explore the reported experiences, difficulties and proposed solutions of chief investigators (CIs) recruiting CYP with LLCs/LTIs and families in the UK.

**Methods:**

61 CIs conducting studies with CYP with LLCs/LTIs and their families, identified from the UK National Institute of Health Research portfolio, completed an anonymous, web-based questionnaire, including both closed and open-ended questions. Descriptive statistics and inductive and deductive coding were used to analyse responses.

**Results:**

UK CIs cited limitations on funding, governance procedures including Research and Development, Site-Specific and REC approval processes, and clinician gatekeeping as challenges to research. CIs offered some solutions to overcome identified barriers such as working with CYP and their families to ensure their needs are adequately considered in study design and communicated to ethics committees; and designing studies with broad inclusion criteria and developing effective relationships with clinicians in order to overcome clinician gatekeeping.

**Conclusions:**

Many of the challenges and solutions reported by UK CIs have applicability beyond the UK setting. The involvement of clinicians, patients and their families at the inception of and throughout paediatric palliative care research studies is essential. Other important strategies include having clinician research champions and increasing the visibility of research. Further research on the perspectives of all stakeholders, leading to mutually agreed guidance, is required if care and treatment are to improve.

## Introduction

In the UK, around 32 per 10 000 children have a life-limiting condition (LLC) or life-threatening illness (LTI).^1^ LLCs are those from which there is no reasonable hope of cure and from which children and young people (CYP) will die. LTIs are those for which curative treatment may be possible but can fail.^1^ CYP with LLCs/LTIs are likely to receive a palliative approach to care, often alongside disease-directed treatments.^2^ In this population, the physical, social and psychological needs of patients and families are often great and may not be well addressed.^3 4^ There is a lack of evidence in paediatric palliative care (PPC) and further research of all types is required.^5–7^

A systematic review exploring recruitment strategies in studies conducted in this field found that invitation and recruitment of participants were under-reported.^8^ Recruitment is challenging at all stages including identification of potential participants, approach and invitation to participate, and the initial consent process.^8–12^

An ever-increasing emphasis on the importance of involving children and their families from the study design stage forward has occurred at a time when there have also been changes in the UK governance and regulation of research.^13^ As of 1 January 2015, the Health Research Authority (HRA) was established to streamline the process of obtaining ethical and regulatory approvals and Research and Development (R&D) approval has changed.^14^

Despite researchers being responsible for navigating these complex approval processes and many chief investigators (CIs) having several years’ experience in the field of palliative care, very little research has directly explored their views about barriers encountered and suggested solutions to overcome these. Chen and colleagues^15^ conducted an interview study of leading researchers in palliative care in the USA in order to explore barriers to research they had encountered. However, this work was not focused exclusively on studies conducted with children. Therefore, we conducted a scoping survey of PPC researchers at the 7th International PPC conference in 2015. This initial survey reported clinician gatekeeping and research ethics committees (RECs) as significant barriers to recruitment in this population and the full results are reported elsewhere.^16^ We used these data to inform the structure and content of a larger and more detailed online national survey to further our understanding.

In this paper, we report on the UK wide online survey which collected data from CIs recruiting CYP with LLCs/LTIs and their families, with attention not only to reported barriers to research but also suggestions for solutions.

## Methods

### Development of the survey

An online survey was built using OPINIO survey software.^17^ As well as building on what we had learnt from the scoping survey of PPC researchers,^16^ the UK wide survey was also informed by a scoping review of barriers to research in palliative care with paediatric and with adult populations.^9–12 15 18–25^ That review identified six relevant domains: (1) ethical issues; (2) institutional capacity; (3) funding issues; (4) physician-related factors; (5) population factors and (6) recruitment and consent processes. The UK wide survey was drafted by the research team which included senior researchers experienced in conducting palliative care research in a number of populations including CYP and their families, as well as more junior researchers who are often responsible for navigating governance procedures and recruiting research participants.

The draft survey was piloted by the research team among both junior and senior academic colleagues, including three statisticians, and was modified in an iterative process based on their feedback. Items were presented in a variety of formats including multiple response, Likert scale and open-ended questions.

The final version of the survey included 28 questions and was divided into three sections collecting data on: (1) CI’s most recent project involving CYP with LLCs/LTIs/their families—including sections on ethical and regulatory approvals and researcher access to patients and families via clinicians; (2) CI’s experiences of research with this population in general; (3) demographic information on respondents. A copy of the survey is included in the online supplementary appendix A.

10.1136/bmjspcare-2018-001521.supp1 Supplementary data

### Participants and setting

The UK National Institute of Health Research (NIHR) Clinical Research Network (CRN) portfolio was searched to identify all CIs conducting studies with CYP (aged 0–18 years) with LLCs/LTIs and their families which were either open or closed (ie, collecting data) between March 2011 and 2016. There were no restrictions on methodology of included studies. Decisions on whether an illness or condition was considered to be LL or LT were guided by Hain’s directory of LLCs^26^ which includes International Classification of Diseases-10 codes. If there was uncertainty about eligibility, CIs were included initially as the survey began by asking whether respondents had been involved with a study that recruited CYP with LLCs/LTIs or their families over the past 5 years and automatically terminated if they selected ‘No’.

All potentially eligible and eligible CIs (n=257) were sent a personalised invitation via e-mail in April 2016 and a reminder was sent 1 month later. The first page of the survey served as an information sheet for respondents, including the project aims, definitions for LLCs/LTIs and approximate time for completion (15 min). Consent to participate was considered implied by voluntary completion of the survey.^27^

### Study design

We used a mixed methods design; qualitative open-ended items were present to validate and expand on the quantitative components.^28^ Closed-ended questions were analysed using descriptive statistics. We analysed responses to open-ended questions using the broad principles of grounded theory, with a focus on inductive coding while acknowledging the use of deductive coding, as appropriate.^29^

### Ethics

Ethical approval was granted from the UCL Research Ethics Committee (UCL ethics Project ID: 8513/001) on 11 March 2016.

## Results

### Sample

Of the 257 CIs invited to take part, 71 answered the first eligibility question on the survey; of these 4 answered ‘No’ to this and 6 answered ‘Yes’ but then answered no further questions. Twenty-four per cent (61/257) of those who were invited to take part, did so beyond the initial eligibility question. Respondent characteristics are shown in table 1.

**Table 1:** Characteristics of CIs

Variable	n (%)
Type of post (n=53)	
Combined (research and clinical)	27 (51)
Research	15 (28)
Clinical	11 (21)
Discipline (n=53)	
Medicine	33 (62)
Nursing	5 (9)
Social science	4 (8)
Allied Healthcare	3 (6)
Education	1 (2)
Natural science	1 (2)
Other	6 (11)
Time spent researching with CYP with LLC/LTI and their families (n=53)	
>16 years	21 (40)
11–15 years	7 (13)
6–10 years	12 (23)
1–5 years	12 (23)
<1 year	1 (2)
Number of studies recruiting CYP with LLC/LTI CI has been involved in (n=52)	
11+	10 (19)
6–10	16 (31)
2–5	11 (21)
1	15 (29)

CI, chief investigator; CYP, children and young people; LLC, life-limiting condition; LTI, life-threatening illness.

### Characteristics of research project for which CI most recently completed data collection

Studies were diverse in nature and key features of these projects are summarised in table 2. CYP in the studies had a range of LLCs/LTIs, including for example cystic fibrosis, cerebral palsy, cancer and Duchenne muscular dystrophy.

**Table 2:** Characteristics of research project for which CIs most recently completed data collection

Variable	N (%)
Type of project (n=61)	
Multicentre	46 (75)
Single site	15 (25)
Methodology (n=61)	
Randomised controlled trial	18 (30)
Observational study	14 (23)
Non-randomised trial	8 (13)
Interviews	7 (11)
Mixed methods study	7 (11)
Questionnaire	4 (7)
Focus group	2 (3)
Participant observation	1 (2)
Use of audio/video/the internet (n=60)	
Audio recording	21 (35)
Video	7 (12)
Internet	7 (12)
Study participants (n=60)	
Children and young people	57 (95)
Parents	27 (45)
Health professionals	19 (32)
Siblings	8 (13)
Language of children and young people and families (n=61)	
English as a second language	44 (72)

CI, chief investigator.

### Barriers to research with CYP with LLC/LTI and their families

#### Ethical and regulatory approvals

For the most recent project for which CIs had completed data collection, 44% stated that they experienced difficulties gaining R&D approval and 26% site-specific approval (SSA). Only 14% said they had difficulties obtaining REC approval (table 3). Respondents emphasised that it was both the complexity and duration of the R&D/SSA approval processes that made these processes challenging.

**Table 3:** Difficulty and changes required for gaining approvals for most recent research project

Variable	N (%)
Whether difficulties were experienced gaining approval (n=56)	
R&D (n=55)	24 (44)
SSA (n=46)	12 (26)
Coordination of approval systems (n=56)	12 (21)
REC (n=56)	8 (14)
CAG (n=5)	1 (20)
Review within institution (n=40)	1 (3)
Other (n=12)	1 (8)
Most difficult approval process (for those experiencing difficulties) (n=32)	
R&D	15 (47)
SSA	7 (22)
Coordination of approval systems	4 (13)
REC	1 (3)
CAG	1 (3)
Review within institution	0 (0)
Other	4 (13)
Whether changes were required as a result of review process (n=56)	
R&D (n=55)	15 (27)
SSA (n=46)	5 (11)
REC (n=56)	35 (63)
CAG (n=5)	2 (40)
Review within institution (n=40)	7 (18)
Other (n=12)	3 (25)
Area where the biggest change was requested (n=40)	
Information sheet	19 (48)
Consent/assent	9 (23)
Data collection methods	4 (10)
Sample size	2 (5)
Data storage	1 (3)
Other	5 (13)

R&D, Research and Development; SSA, Site Specific Approval; REC, Research Ethics Committee; CAG, Confidentiality Advisory Group.

Notably, 63% of CIs reported having to make changes after REC review when compared with 27% of CIs after R&D review (table 3). Forty-five per cent (18/40) of CIs found the changes to study conduct and materials required by regulators to be useful. For 45% (25/56) of respondents, it took >6 months to obtain all necessary approvals.

#### Researcher access to patients and families via clinicians

For the most recent project for which CIs had completed data collection, 32% (17/53) of respondents rated the working relationship between clinicians and researchers as extremely effective and 51% (27/53) as effective. However, such a positive assessment was not mirrored in free text responses, where CIs emphasised how the quality of working relationships could vary by individual, team and centre. Variability in clinicians’ willingness to recruit participants was particularly highlighted, as illustrated by the comment below:

Some clinicians act as gatekeepers and do not give families the opportunity to decline or accept a study (CI 10, researcher).

#### Funding

**Figure 1:**
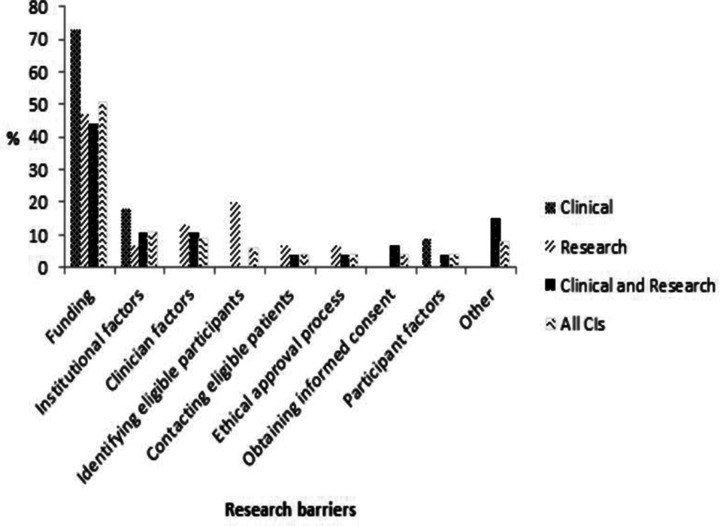
CIs views of the biggest barriers to research with CYP with LLCs/LTIs and their families. CI, chief investigator; CYP, children and young people; LLC, life-limiting condition; LTI, life-threatening illness.

Regarding their experiences in research with CYP with LLCs/LTIs and their families in general, the majority of CIs (51%; 27/53) reported that funding was the biggest research barrier encountered. This was especially true for those in a clinical (73%; 8/11) rather than a clinical and research (44%; 12/27) or solely research (47%; 7/15) position (figure 1).

### Respondents’ proposed solutions to identified barriers and challenges to research with CYP with LLC/LTI and their families

Respondents proposed recommendations that are likely to be applicable, regardless of the population under study. These included having a well thought out question and methodology to improve chances of obtaining funding, attending ethics meetings in person and getting advice from R&D, RECs and others who have been through the approval process. There were also a number of recommendations that related specifically to PPC research. These are detailed below:

#### Involving CYP and their families throughout the research process

The importance of involving CYP and families early in the research process was strongly indicated. Ensuring that ‘the study design adequately takes into consideration the participant families’ needs’ (CI15, research and clinician) was emphasised. Many CIs spoke about the importance of involving CYP in the design of clear and appropriate participant information sheets. Written information sheets might not be the most appropriate, as reflected by the comment from one CI below:

Our population had a lot of parents who either didn't read and write in English or who had limited literacy in any language. I understand this to be common to many palliative conditions. In practice the written information on consent sheets and information sheets were not helpful in the slightest (CI 17, researcher).

Respondents underscored how feedback from CYP and their families must be included in ethics applications:

Consult with members of the communities you plan to involve… Record their opinions and include them in your application for ethical approval (CI 20, researcher).

#### Designing studies with broad inclusion criteria

Respondents indicated that given the usually small number of eligible CYP for PPC research, inclusion criteria should be broad to ensure a large enough sample size is reached:

Design studies that will not exclude too many patients. This is very important in children (CI 33, clinician).

#### Involving clinicians early on in the research process

It was argued that clinicians should be involved in the design, development and conduct of research from the outset:

Make sure you establish excellent relationships with clinicians and parent and patient groups well in advance of beginning your research (CI 27, researcher and clinician).

#### Embedding researchers into clinical teams

Seventy-five per cent of CIs (40/53) reported that embedding researchers into clinical teams would have a major impact on improving researcher access to patients and families. However, the challenges in this approach were also noted. As one CI stated:

The medical staff in my 'team' have NO interest in anything other than Clinical Trials of an Investigational Medicinal Product (CTIMPs) (CI 26, researcher and clinician).

One CI suggested that to overcome clinician gatekeeping researchers should be permitted to recruit for themselves:

Interestingly, parents always wanted to participate…I would suggest all researchers should recruit for themselves and not allow clinicians to facilitate it at all as it can cause unnecessary gate-keeping (CI 17, researcher).

However, as highlighted by one CI, this is problematic where recruiting via clinicians is ‘an ethical requirement of the study’ (CI 19, researcher and clinician).

## Discussion

We undertook this work to increase our understanding of current barriers to conducting research with CYP with LLCs/LTIs and their families, and to consider how these might be overcome. Our data reflect the views of CIs conducting studies in the UK with funds from NIHR or organisations that meet quality criteria for inclusion in the NIHR Portfolio (eg, Wellcome Trust, Cancer Research UK, The Health Foundation). As emphasised by the results, particular challenges arise in PPC research: opportunities for funding are scarcer both through mainstream agencies and specific calls, RECs may be apprehensive about approving work in this area and overloaded clinicians may also have anxieties about the appropriateness of some research work. CIs emphasised the importance of involving clinicians, CYP and families at the inception of studies and embedding researchers into clinical teams, in order to overcome some of the identified difficulties.

### What is already known about this topic and what this research/review adds

The findings of this study in the UK are consistent with others exploring similar issues such as that by Chen *et al* reporting on results of a survey of US National Institute of Health funded CIs.^15^ This underscores how regardless of differences between countries and healthcare systems, problems encountered in this area are common. Unique to our study was the challenge that CIs raised with regard to the R&D approval process. It is unclear, however, whether these perceived difficulties are specific to PPC research and the extent to which such obstacles will have persisted since the establishment of the HRA and streamlining of processes in the UK in 2015. This is something that would be an important area for further study.

Also, unique to our study was the fact that CIs were explicitly asked to propose solutions to overcome barriers. The stated importance of co-designing research projects from the outset with CYP and their families is in line with policy and is of particular importance for PPC research to address the concerns of RECs and clinicians who can have distorted assumptions about families’ views about potential participation.^8 12 30 31^ Furthermore, it was underlined by CIs how the biggest changes were required to information sheets and then the consent/assent process before approvals were granted and, consequently, the importance of involving CYP in the design of information sheets was specifically mentioned. It was argued that written information sheets might not be the most appropriate for the PPC population. Although focused on decision making for participation in healthcare trials involving CYP (the benefits and burden of participation are likely to differ for non-trial studies), a current trial is underway to evaluate how multimedia interventions, including textual, audio and visual information compare with written information.^32^

The involvement of clinicians from the study design stage was viewed as important by CIs and should be considered a priority in future guidance. Taking a participatory approach to research, with health professionals involved early on, has been shown to be effective in prior palliative care research,^33^ both in terms of the representativeness of the sample and positive perspective of health professionals when collecting the data.^33^

The perceived importance of embedding researchers into clinical teams to improve access to patients and families is in line with the experiences of Bluebond-Langner and colleagues, who embedded researchers in a longitudinal study and enabled different perspectives of multiple participants to be captured in real time.^34^

However, as emphasised by CIs in our survey, even if clinicians are involved early on in the research process and researchers are embedded into clinical teams, recruitment via clinicians can still be a challenge.^35^ A review of interventions conducted to improve recruitment to randomised controlled trials found that the impact of interventions aimed at recruiters, specifically providing additional education, was unclear.^36^ We suggest the development of clinician research champions to facilitate research within their teams. When reporting work carried out with the active co-operation of clinicians, these individuals should be invited to contribute as full authors for peer-reveiwed manuscripts or at least be acknowledged within these. Use of research registers, publicising research on social media or university websites, and liaising with special interest groups for rarer LLCs and LTIs are possible mechanisms to increase the profile and visibility of research within society, so interested families can request to participate in studies. A further important strategy, adopted as part of a participatory approach to research in this study and in other research areas,^37^ is ensuring that there is a lay representative on the research team.

### Strengths and limitations of the study

The use of the NIHR portfolio to identify CIs meant that the scope of the survey could be nationwide. However, a ‘true’ population denominator could not be calculated as it was sometimes difficult to assess whether some CIs should have been included, for example, where there was limited information about the condition studied.

By only using the NIHR portfolio, the perspectives of those who conducted studies without NIHR or NIHR recognised funding sources (eg, studies funded by some approved charities)^38^ were not included. The use of the portfolio to identify participants also meant that clinicians were likely to be over-represented in the sample. However, the number of highly experienced researchers in the study (n=61) provided a rich source of data for analysis.

Conducting a survey that included both closed and open-ended questions provided depth and clarity in responses. This was particularly striking where CIs described clinician involvement with introducing the study to patients/families as positive in a closed question but in open-ended responses, variability in clinicians’ willingness to recruit participants to studies was highlighted.

### Directions for further research

Our survey was conducted from the perspectives of CIs. There remains little in the literature on what other clinicians, parents and CYP perceive as barriers to PPC research and how these might be overcome. Seeking the perspectives of funding bodies would also be useful. However, identifying suitable interventions in this area is likely to be complex. Informed by the findings of this research, our group, in collaboration with the HRA, is now conducting a more in-depth study of the constraints and concerns of RECs with the goal of reducing barriers inherent in current research governance processes.^39^
