# Supplementary material for: Research barriers in children and young people with life-limiting conditions: a survey
Source: BMJ Support Palliat Care. 2018 Jul 31;12(e5):e715–21. doi: 10.1136/bmjspcare-2018-001521 (PMC9606545; doi:10.1136/bmjspcare-2018-001521)
Supplement: Supplementary data [file bmjspcare-2018-001521supp001.pdf]

Barriers to Research Access: Voices, Experiences and Solutions

Your experiences of conducting research with children and young people ages 0-18 with life-limiting conditions and life-threatening illnesses

This survey will take approximately 15 minutes to complete.

This survey is the first step in a project that aims to improve the access to and participation of children and young people (CYP) with life-limiting conditions and life-threatening illnesses (LLC-LTI) and their families in research. We aim to understand, and where necessary, change the behaviour of clinicians and Research Ethics Committees (REC) to further enable the research process. This is your opportunity to have your say and be a part of the process.

The survey contains three short sections:

Section 1: This section contains questions on: a) Characteristics of the research project for which you have most recently completed data collection, b) Ethical and regulatory approvals for that study and c) Researcher access to patients and families for that study.

Section 2: Your experience of research with CYP and their families *in general.*

Section 3: A very brief demographic section.

Voluntary nature of participation and anonymity

Completion of the survey stands as your consent to participate.

However, your participation in the study is entirely voluntary and you are under no obligation to take part and are free to withdraw from the study at any time, without giving a reason for doing so. You do not have to answer any questions in the survey that you do not want to.

The data that you do provide will be completely anonymous. The results from the survey will be reported in a scholarly journal.

Contact details for further information

Should you have any further queries about the survey the contact details of the study coordinator and principle investigator are below:

Study coordinator: Jordana Peake j.peake@ucl.ac.uk

Principle investigator: Professor Myra Bluebond-Langner bluebond@ucl.ac.uk

We thank you for taking the time to complete the survey

Life-limiting conditions are those for which there is no reasonable hope of cure and from which children or young people will die (Together for short lives definitions for professionals, accessed from http://www.togetherforshortlives.org.uk/professionals/childrens_palliative_care_essentials/definitions)

Life-threatening illnesses are those for which curative treatment may be feasible but can fail (ACT/RCPCH. A guide to the development of children's palliative care services: report of the joint working party. Bristol, 1997.)

Peake JN, *et al. BMJ Support Palliat Care* 2022; 12:1–7. doi: 10.1136/bmjspcare-2018-001521

Section 1 - Your research project

A Characteristics of the research project

Please answer the following questions in relation to the research project for which you have most recently completed data collection that actively recruited children and young people (CYP) with life-limiting conditions or life-threatening illnesses (LLC-LTIs)/their families.

Q1: Have you been involved with a study actively recruiting CYP with LLC-LTIs or their families in the past five years?

◯ Yes ◯ No (This will automatically end the survey)

If your research is not with CYP with LLC-LTIs, you do not need to complete this survey, selecting No will automatically end the survey.
If you are unsure about whether the CYP in your study had LLC-LTIs please select Yes and complete the survey (your answer to later questions will help us to determine if your population is life-limited or life-threatened).

Note: if you have answered/chosen item [2] in question 1, skip the following question

Q2: In which of the following institutions are you based? Please select Yes or No for each category.

	Yes	No
University	◯	◯
Hospital	◯	◯
Other clinical setting e.g. hospice	◯	◯

Other (please state)

Note: if you have answered/chosen item [2] in question 1, skip the following question

Q3: Which of the following best describes the methodology you used? Please select one category which best describes your study.

◯ Randomised controlled trial
◯ Non randomised trial
◯ Focus group
◯ Interviews
◯ Participant observation
◯ Questionnaire
◯ Observational study
◯ Mixed methods study (please state)

If you have chosen "other", please specify:

Note: if you have answered/chosen item [2] in question 1, skip the following question

Q4: Which of the following groups are in your population? Please select Yes or No for each category.

	Yes	No
Infants (<1 year of age)	◯	◯
Children (1-12 years old)	◯	◯
Young people (13-18 years old)	◯	◯
Parents	◯	◯
Siblings	◯	◯
Healthcare professionals	◯	◯

Other (please state)

Note: if you have answered/chosen item [2] in question 1, skip the following question

Q5: Which of the following best describes the CYP your study is about? Please select one category.

◯ CYP alive at recruitment
◯ CYP deceased at recruitment
◯ Mixed (some CYP alive at recruitment and some deceased CYP at recruitment)
◯ Other (please state)

If you have chosen "other", please specify:

Note: if you have answered/chosen item [2] in question 1, skip the following question

Q6: Did any of your methodologies use audio recording, video or the internet to collect data? Please select Yes or No for all categories.

Peake JN, *et al. BMJ Support Palliat Care* 2022; 12:1–7. doi: 10.1136/bmjspcare-2018-001521

	Yes	No
Audio recording	◯	◯
Video	◯	◯
Internet	◯	◯

Note: if you have answered/chosen item [2] in question 1, skip the following question

Q7: Did you include CYP and families with English as a second language in your project?

◯ Yes ◯ No

Note: if you have answered/chosen item [2] in question 1, skip the following question

Q8: Is your research project a multi-centre project?

◯ Yes ◯ No

Note: if you have answered/chosen item [2] in question 1, skip the following question

Q9: What are the diagnoses of the CYP your study is looking at? Please state.

[ ]

B Ethical and regulatory approvals

Please answer the following questions in relation to the research project for which you have most recently completed data collection that actively recruited children and young people (CYP) with life-limiting conditions or life-threatening illnesses (LLC-LTIs)/their families.

Note: if you have answered/chosen item [2] in question 1, skip the following question

Q10: Did you need to receive approval from the following regulatory bodies/procedures for your study? Please select Yes or No for each category.

	Yes	No
NHS Research Ethics Committee (REC)	◯	◯
Research and Development (R&D)	◯	◯
Site-Specific Assessment (SSA)	◯	◯
Review within your institution	◯	◯
Confidentiality Advisory Group (CAG)	◯	◯
Other (please specify in the box below)	◯	◯

Peake JN, *et al. BMJ Support Palliat Care* 2022; 12:1–7. doi: 10.1136/bmjspcare-2018-001521

Other

Note: if you have answered/chosen item [2] in question 1, skip the following question
Note: if you have answered/chosen all of the following items: [(3, 2), (3, 3), (3, 4), (3, 5), (3, 6), (3, 7)] in question 10, skip the following question

Q11: How long (approximately) did it take you in total to receive approval from all the relevant regulatory bodies/procedures (from filling out the appropriate forms to gaining final permissions)? Please select one time period.

◯ 0-3 months ◯ 4-6 months ◯ 7-9 months ◯ 10-12 months ◯ Over 12 months

Note: if you have answered/chosen item [2] in question 1, skip the following question
Note: if you have answered/chosen all of the following items: [(3, 2), (3, 3), (3, 4), (3, 5), (3, 6), (3, 7)] in question 10, skip the following question

Q12: Did you experience difficulty gaining approval for any of the following regulatory bodies/procedures? Please select Yes or No for each category.

	Yes	No
NHS Research Ethics Committee (REC)	◯	◯
Research and Development (R&D)	◯	◯
Site-Specific Assessment (SSA)	◯	◯
Review within your institution	◯	◯
Confidentiality Advisory Group (CAG)	◯	◯
Coordination of approval systems	◯	◯
Other (Please specify below)	◯	◯

Other

Peake JN, *et al. BMJ Support Palliat Care* 2022; 12:1–7. doi: 10.1136/bmjspcare-2018-001521

Note: if you have answered/chosen item [2] in question 1, skip the following question
Note: if you have answered/chosen all of the following items: [(3, 2), (3, 3), (3, 4), (3, 5), (3, 6), (3, 7)] in question 10, skip the following question
Note: if you have answered/chosen all of the following items: [(3, 2), (3, 3), (3, 4), (3, 5), (3, 6), (3, 7), (3, 8)] in question 12, skip the following question

Q13: If you experienced difficulties during the approval process, which part was the most difficult? Please select one body/procedure.

- ○ NHS Research Ethics committee (REC)
- ○ Research and Development (R&D)
- ○ Site-Specific Assessment (SSA)
- ○ Review within your institution
- ○ Confidentiality Advisory Group (CAG)
- ○ Coordination of approval systems
- ○ No difficulties experienced
- ○ Other (please state)

If you have chosen "other", please specify:

Note: if you have answered/chosen item [2] in question 1, skip the following question
Note: if you have answered/chosen all of the following items: [(3, 2), (3, 3), (3, 4), (3, 5), (3, 6), (3, 7)] in question 10, skip the following question

Q14: Were you required to make any changes as a result of review of the project by the following regulatory bodies/procedures?
Please select Yes or No for each category.

	Yes	No
NHS Research Ethics Committee (REC)	○	○
Research and Development (R&D)	○	○
Site-Specific Assessment (SSA)	○	○
Review within your institution	○	○
Confidentiality Advisory Group (CAG)	○	○
Other (please specify below)	○	○

Other

Peake JN, *et al. BMJ Support Palliat Care* 2022; 12:1–7. doi: 10.1136/bmjspcare-2018-001521

Note: if you have answered/chosen item [2] in question 1, skip the following question
Note: if you have answered/chosen all of the following items: [(3, 2), (3, 3), (3, 4), (3, 5), (3, 6), (3, 7)] in question 10, skip the following question
Note: if you have answered/chosen all of the following items: [(3, 2), (3, 3), (3, 4), (3, 5), (3, 6), (3, 7)] in question 14, skip the following question

Q15: If you were required to make changes in your applications before approval was granted, what was the biggest change that was requested? Please select the one category which the main changes addressed.

◯ Study sample size

◯ Consent process with parents

◯ Consent process with young people

◯ Assent process with children

◯ Consent form for parents

◯ Consent form for young people

◯ Assent form for children

◯ Information sheet for parents

◯ Information sheet for young people

◯ Information sheet for children

◯ Methods of data collection (e.g. survey, tissue and blood samples, interviews, participant observation, focus group)

◯ Data storage

◯ The data analysis (e.g. statistical analysis, tissue analysis, thematic analysis, grounded theory analysis, conversational analysis)

◯ Other (please state)

If you have chosen "other", please specify:

Note: if you have answered/chosen item [2] in question 1, skip the following question
Note: if you have answered/chosen all of the following items: [(3, 2), (3, 3), (3, 4), (3, 5), (3, 6), (3, 7)] in question 10, skip the following question
Note: if you have answered/chosen all of the following items: [(3, 2), (3, 3), (3, 4), (3, 5), (3, 6), (3, 7)] in question 14, skip the following question

Q16: Did you find the changes required for project approval useful? Please rate the usefulness on the scale below.

Peake JN, *et al. BMJ Support Palliat Care* 2022; 12:1–7. doi: 10.1136/bmjspcare-2018-001521

- ◯ Very Useful
- ◯ Useful
- ◯ Neither useful nor unuseful
- ◯ Unuseful
- ◯ Very Unuseful
- ◯ N/A

Note: if you have answered/chosen item [2] in question 1, skip the following question
Note: if you have answered/chosen all of the following items: [(3, 2), (3, 3), (3, 4), (3, 5), (3, 6), (3, 7)] in question 10, skip the following question

Q17: What recommendations would you give to other investigators to facilitate the approval process?Please state.

C Researcher access to patients and families via clinicians

Please answer the following questions in relation to the research project for which you have most recently completed data collection that actively recruited children and young people (CYP) with life-limiting conditions or life-threatening illnesses (LLC-LTIs)/their families.

Note: if you have answered/chosen item [2] in question 1, skip the following question

Q18: What was your experience of involving the patients clinician in each of the following parts of the research process? Please rate overall experience for each of the research tasks.

Research Task	Very positive	Positive	Neither positive nor negative	Negative	Very negative	N/A
Identifying the patients	◯	◯	◯	◯	◯	◯
Introducing study to patients/families	◯	◯	◯	◯	◯	◯
Gaining consent from the patients/families	◯	◯	◯	◯	◯	◯
Being participants in the project themselves (e.g. being observed)	◯	◯	◯	◯	◯	◯
Other (please state)	◯	◯	◯	◯	◯	◯

Note: if you have answered/chosen item [2] in question 1, skip the following question

Q19: How would you describe the working relationship between researchers and clinicians in your study? Please rate your overall experience.

- ◯ Extremely effective
- ◯ Effective
- ◯ Neither effective nor ineffective
- ◯ Slightly ineffective
- ◯ Not at all effective
- ◯ N/A

Note: if you have answered/chosen item [2] in question 1, skip the following question

Q20: If you would like to comment on your answer to the above question, please do so here:

Note: if you have answered/chosen item [2] in question 1, skip the following question

Q21: The following interventions have been suggested to improve researcher access to patients and families. Please indicate the likely impact of each of the suggested interventions.

Intervention	Large impact	Moderate impact	Neutral	Low impact	No impact
Increase clinician's awareness of the research before it starts	◯	◯	◯	◯	◯
Include clinicians in development of research questions in projects	◯	◯	◯	◯	◯
Provide time for researchers and clinicians to spend discussing aspects of the research	◯	◯	◯	◯	◯
Provide greater dissemination of project findings which show benefit to patient care in general amongst clinicians	◯	◯	◯	◯	◯

Peake JN, *et al. BMJ Support Palliat Care* 2022; 12:1–7. doi: 10.1136/bmjspcare-2018-001521

Provide for frequent discussions between researchers and clinicians about progression on the project and results	○	○	○	○	○
Embed researchers into clinical teams	○	○	○	○	○

Please state any other interventions you think would be useful in improving researcher access via clinicians:

Section 2 –Research with children and young people with life-limiting conditions or life-threatening illnesses (LLC-LTIs).

This section refers to your experiences in research with CYP with LLC-LTI/their families in general

Note: if you have answered/chosen item [2] in question 1, skip the following question

Q22: How many studies involving the recruitment of children with LLC-LTI have you been involved in? (i.e. directly recruiting the children or overseeing the recruitment process)?

○ 1 ○ 2-5 ○ 6-10 ○ 11+

Note: if you have answered/chosen item [2] in question 1, skip the following question

Q23: Thinking about your research with CYP with LLC-LTI and their families in general, what do you find is the biggest challenge in conducting your research? Please pick the one option you see as the biggest barrier from the list below or please state your own if not on the list.

○ Funding
○ Participant factors
○ Obtaining informed consent
○ Other (please state)
○ Institutional factors
○ Identifying eligible participants
○ Attrition
○ Ethical approval process
○ Contacting eligible patients
○ Clinician factors

If you have chosen "other", please specify:

Note: if you have answered/chosen item [2] in question 1, skip the following question

Q24: What would you recommend to other investigators to facilitate research with this population? Please state.

Section 3 - Please answer a few questions about yourself

Note: if you have answered/chosen item [2] in question 1, skip the following question

Q25: What is the main discipline you work in?Please select the one discpline you see as your main area of work from the disciplines below.

- ◯ Allied Health Care
- ◯ Education
- ◯ Formal Science (e.g. Maths, Statistics)
- ◯ Medicine
- ◯ Nursing
- ◯ Natural Science (e.g. Biology, Chemistry)
- ◯ Social Science (e.g. Anthropology, Sociology, Psychology)
- ◯ Other (please state)

If you have chosen "other", please specify:

Note: if you have answered/chosen item [2] in question 1, skip the following question

Q26: Is your post clinical, research or combined?Please select one option from the list below.

◯ Clinical ◯ Research ◯ Combined

Note: if you have answered/chosen item [2] in question 1, skip the following question

Q27: Approximately, how long have you been involved in research with children and young people with LLC-LTI or their families? Please select one option.

◯ Less than 1 year ◯ 1-5 years ◯ 6-10 years ◯ 11-15 years ◯ Over 16 years

Note: if you have answered/chosen item [2] in question 1, skip the following question

Q28: Thank you for taking the time to complete this questionnaire. If you have any further comments regarding your experiences of research with children and young people with LLC-LTI and their families, please write them below.

Peake JN, *et al. BMJ Support Palliat Care* 2022; 12:1–7. doi: 10.1136/bmjspcare-2018-001521
